# The Chigoe, or Gigger

**Published:** 1843-04

**Authors:** Frederick Roberts

**Affiliations:** Assistant Surgeon of the 59th Regiment


*The Chigoe, or Gigger.* By Frederick Roberts, M. D., Assistant Surgeon of the 59th Regiment.—As medical authors give no account of the chigoe, which becomes so often parasitic to man, and a source of considerable annoyance to him in the West Indies, and as it has further been doubted to be an insect, the following short paper, containing the slender information that could be gathered from a few examinations of cases, respecting the habits, effects on the body of man, and treatment for its removal, is offered.

The "chigoe," or "chiego," of the West Indies, and the "pulex penetrans" of naturalists, consists of two species, the black and the white, in the natural history of which there appears to be no difference. The habitation of the "gigger," as it is likewise called, is in stables, kitchens, and on the ground in the open air in dry weather, which is the season it mostly prevails in—that is, from December to July. It attaches itself to the feet of those who walk about bare-footed, and those whose occupations are in kitchens, abounding in ashes and other filth. Holes in boots or shoes render one liable to be attacked by them. Its intrusions are not confined to the human species, but it is found to attach itself to cats, dogs, sheep, and still more to pigs.

The parts of the body it is found in are the soles of the feet and toes, and occasionally the fingers. It insinuates itself beneath the skin, and there deposits its eggs in a nidus of a tough gelatinous substance. Attention is first drawn to the presence of these insects by a gentle itching, which, as the insect insinuates itself deeper, and the nidus begins to grow, becomes, in a few days, less tolerable. The nidus, containing longish-shaped white gelatinous eggs, is a cyst of a tough membrane, and arrives sometimes to the size of a pea before the eggs are hatched, after which the new being likewise insinuates itself into the skin; so that in those who neglect extracting them there are clusters, buried under the skin of the soles of the feet and toes, which raise it in little round eminences, and create irritable sores, oozing a serous fluid, and ultimately intractable ulcers—nay, the entire toe has been known to be lost by the creeping ulceration that is established. The black gigger differs so much in its effects from the white, to which, it has been said, it otherwise resembles, as to be called the poisonous gigger, and produces much more malignant sores. The treatment for the removal of the chigoe consists in extraction by means of a needle—an operation dexterously performed by the negroes, and free from pain.—*New York Lancet*, from *London Med. Gazette.*

